# Infrared Absorption Spectrum of Nitrous Oxide (N_2_O) From 1830 cm^−1^ to 2270 cm^−1^*

**DOI:** 10.6028/jres.068A.006

**Published:** 1964-02-01

**Authors:** Earle K. Plyler, Eugene D. Tidwell, Arthur G. Maki

## Abstract

The frequencies of the vibration-rotation spectrum of N_2_O have been measured from 1830 cm^−1^ to 2270 cm^−1^. A number of weak bands have been measured and assigned to “hot bands’’ and isotopic species in normal abundance. By using the Ritz principle and previously measured bands the bending frequency (*v*_2_) is calculated as 588.78_0_ cm^−1^. Frequencies are given for lines arising from the three principal transitions found in this region.

## 1. Introduction

Recently there has been considerable interest in obtaining accurate values for the vibration-rotation potential constants for small molecules. Pliva [1]^1^ has measured the spectra of various isotopic species of nitrous oxide (N_2_O) in the hope of obtaining more data with which to check the anharmonic terms of a potential function which he has devised [2]. Tidwell, Plyler, and Benedict [3] have reported measurements on a large number of vibrational-energy levels for N_2_O and have derived a set of vibration-rotation constants.

Rank et al. [4] have reported the results of some very precise measurements on five absorption bands of N_2_O. McCubbin, Grosso, and Mangus [5] have made some further precise measurements on N_2_O which will be reported soon. While this work was in progress Fraley, Brim, and Rao [6] published results of measurements on the strongest absorption lines due to N_2_O in the 5-*μ* region. The latter measurements are in essential agreement with those reported here.

## 2. Experimental Procedure

The spectra were measured on the NBS high-resolution infrared spectrometer described elsewhere [7]. Most of the measurements were made using a 7,500 lines/in. grating although some measurements were obtained with a 1,860 lines/in. grating. A liquid-nitrogen cooled PbSe detector was used. Calibration was achieved by the combination of accurately measured rare-gas spectra and a Fabry-Perot interferometer fringe system in the manner described in reference 8.

**Figure 1:**
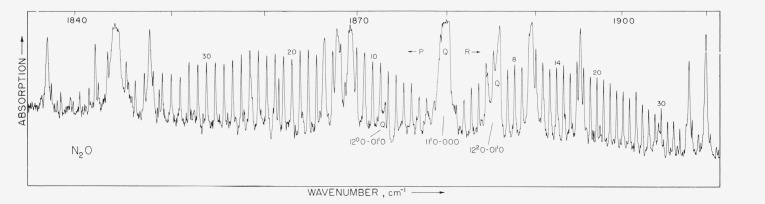
N_2_O *absorption from 1840 to 1910 cm*^−1^. Pathlength 1.2 m; pressure 28 cm Hg.

**Figure 2:**
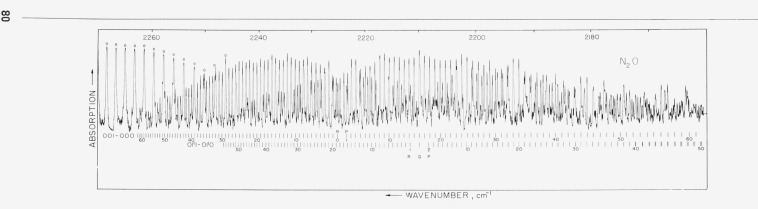
N_2_O *absorption from 2270 to 2160 cm*^−1^. Pathlength 4 m; pressure 1 mm Hg. Circle: indicates absorption lines due to atmospheric C^13^O_2_^16^ in the optical path.

**Figure 3:**
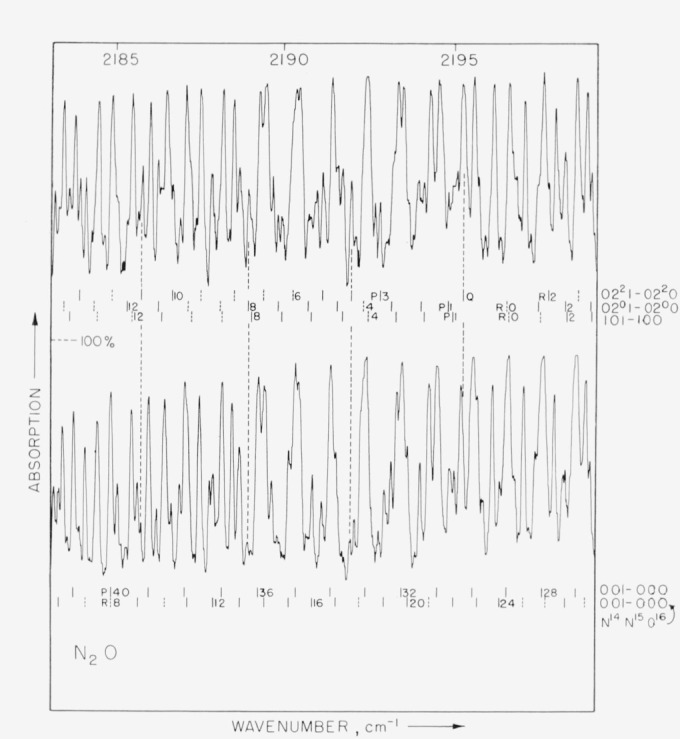
N_2_O *absorption from 2184 to 2200 cm*^−1^. Upper curve is for a temperature of 400 °K and lower curve is with cell cooled to about 220 °K. Pathlength is 1.2 m; pressure 1 mm Hg.

**Figure 4:**
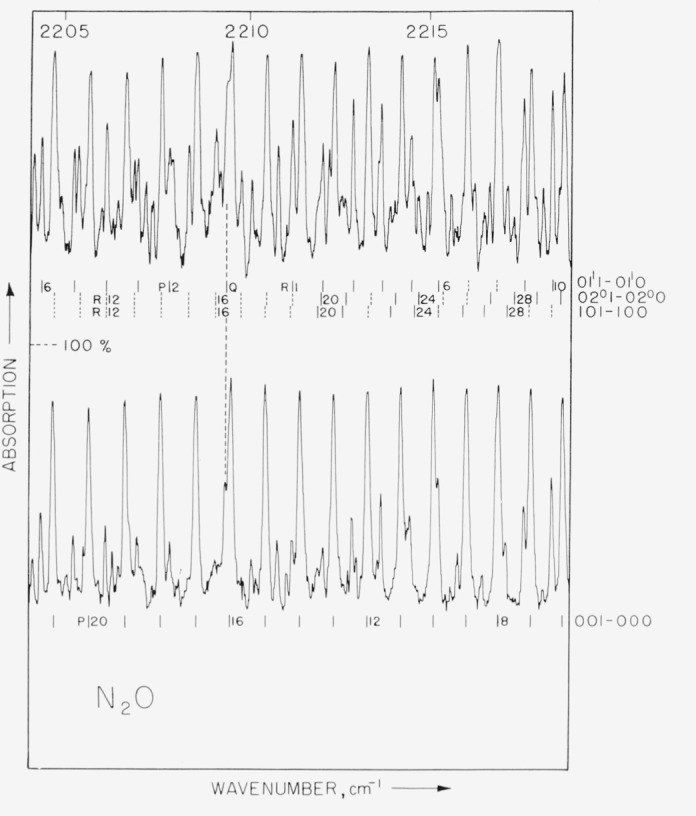
N_2_O *absorption from 2204 to 2220 cm*^−1^. Temperature of gas in upper eurve is 400 °K, temperature of gas in lower curve is 220 °K. Pathlength is 1.2 m; pressure is 2 mm Hg.

Spectra were obtained with pathlengths of 1.2, 4, and 24 m and pressures ranging from 1 to 200 mm Hg. Representative spectra are shown in figures 1 and 2. The 1.2 m cell could be either cooled to 220 °K or warmed to 400 °K; representative spectra obtained at these temperature extremes are shown in figures 3 and 4. In these figures it is evident that many lines which are weak at low temperatures have intensified with increase in temperature. These lines must be attributed to transitions originating from excited vibrational states and accordingly have been assigned as “hot band” lines.

## 3. Analysis of Data

The microwave measurements of Burrus and Gordy [9] have given very precise values for the ground-state rotational constant, *B*_0_, and the *l*-doubling constant, *q*, of the molecule N^14^N^14^O^16^. Coles and Hughes [10] and Coles, Good, and Lide [11] have made further measurements from which one can obtain *B*_0_ for the isotopes N^14^N^15^O^16^, N^15^N^14^O^16^, and N^14^N^14^O^18^. Combining the results of these workers with the velocity of light (taken as 299,793 km/s) we have calculated these constants in wavenumbers as given in table 1. Since these molecular constants are more accurate than could be obtained from the measurements reported here, the values given in table 1 were used wherever applicable for the calculation of the other molecular constants. For this same purpose the value of *D*_0_ = 17.6×10^−8^ cm^−1^ given by Rank et al. [4] was used.

Since the data for many of the bands reported here was rather fragmentary due to the high degree of overlapping, all of the absorption bands were analyzed by obtaining a least-squares fit to the polynomial

$$v_{\text{obs}}=v_{o}+(B^{\prime }+B^{\prime \prime })m+(B^{\prime }-B^{\prime \prime })m^{2}-2(D^{\prime }+D^{\prime \prime })m^{3}-(D^{\prime }-D^{\prime \prime })m^{4}$$

where the terms have their usual significance.

The data were also analyzed using the method of combinations and differences. The results of both methods were comparable, but in many cases the fact that more lines could be used in fitting to the polynomial given above resulted in a reduction in the uncertainty of the various unknown constants when this method was used, especially since the lower state constants were in most cases very accurately known.

For those bands which are split into resolved *c* and *d* components the two bands were analyzed simultaneously to obtain a least-squares fit to the same band center and other constants were made compatible. A more detailed description is given for the individual bands in the next section.

## 4. Results

### 4.1. The Region From 1830 to 1925 cm^−1^

In this region lines have been identified due to the three transitions 12°0–01^l^*^c^*0, 12^2^0–01^1^0, and 11^1^*^c^*0– 000. For all these perpendicular bands the *Q* branches were observed, but the resolution was not good enough to measure any individual *Q* branch lines. The splitting of the Δ—II band was observed for all but a few low *J* lines. The analysis of this Δ—II band was carried out by analyzing the *c* and *d* components simultaneously. Since the data were quite fragmentary for these weak “hot bands,” the best available estimates of the values of $B_{c}^{\prime \prime }$, $B_{d}^{\prime \prime }$, *D″*, $D_{c}^{\prime }$, and $D_{d}^{\prime }$ were used in order to obtain more accurate values of *v*_0_ and *B*′. For this purpose it was assumed that $B_{c}^{\prime }=B_{d}^{\prime }$. Since the *c* and *d* levels of the 12^2^0 state undergo *l*-type resonance with different levels, it was necessary to use different values of *D* for the *c* and *d* levels. The values of the constants used are given in table 2. Only the *c* component of the *v*_1_ *+ v*_2_ band was measured, therefore the value obtained for Δ*B* as given in table 2 is for the transition to the *c* level only.

The calculated and observed frequencies of absorption lines due to the transition 11^1^*^c^*0–000 are given in table 3. Figure 1 shows the appearance of the absorption in this region.

### 4.2. Absorption Lines in the Region 1925 to 2000 cm^−1^

The main band found in this region is a Σ–II band due to the transition 200–01^1^0. In this case the *Q* branch was sufficiently well resolved so that measurements were obtained for both the *c* and *d* levels. Since microwave values for the lower state are quite good, these values were used in the analysis and transitions from both the *c* and *d* levels were analyzed simultaneously by a least-squares program in order to obtain the best values for *v*_0_ and *B*′.

Absorption in this region was very weak as might be expected from the transitions involved. The *Q* branches for the two “hot bands” 21^1^0–02^2^0 and 21^1^0–02^0^0 were also observed in this wavelength region, but they were not resolved. Nor were any lines of the *P* and *R* branches observed.

The calculated and observed frequencies of the lines for the 200–01^1^0 transition are given in table 3.

### 4.3. N^14^N^14^O^16^ Absorption Between 2130 and 2270 cm^−1^

The fundamental *v*_3_ and associated “hot bands” are located in this region. *v*_3_ is a rather strong absorption band, consequently with the pathlength and resolution available it was possible to obtain measurements on four “hot bands” and four isotopic bands.

The splitting of the first “hot band” was observed for high-*J* levels but overlapping with various other bands was rather severe. For this reason it was felt that more accurate band constants could be obtained by averaging the frequencies of the *c* and *d* components. Even though this procedure did not permit the use of measurements where only one component was observed, the resultant constants are believed to be more reliable than those found by analyzing each band individually.

Lines due to the Δ—Δ transition 02^2^1–02^2^0 have been observed and the position of the *Q*, while overlapped, has been verified by observations at 220 °K and 400 °K. Figure 3 shows a few lines due to this transition and the manner in which the line intensities change with temperature. The Boltzman distribution predicts this transition will show an approximate six-fold increase in intensity in going from 220 °K to 400 °K.

Since the two Σ—Σ transitions 101–100 and 02^0^1–02^0^0 are predicted to lie quite close to each other, some difficulty was anticipated in assigning the two series of lines which must be due to these transitions. The assignments of these lines are, however, considered to be reliable due to the rather large differences in the lower-state *B* values. Δ_2_*F*″ plots for these two bands yield respective *B*″ values of 0.4175 and 0.4200 cm^−1^. The *B*″ values expected from the data of reference 3 are 0.41725 and 0.41991, respectively. Many of the low-*J* lines are badly overlapped because the two band centers lie so close together. As a consequence the band centers calculated from the data may be in error by several hundredths of a wavenumber. The statistical treatment of the data resulted in a standard deviation for the *v*_0_ values of 0.01 cm^−1^, but inspection of the data leads us to believe that this is not a realistic number. Therefore table 2 contains a more subjective evaluation of the accuracy of the band centers for these two bands.

Table 4 lists the frequencies of the *v*_3_ band as observed in this laboratory and as reported by Fraley, Brim, and Rao. Columns 3 and 6 compare the calculated frequencies with those observed.

### 4.4. Isotopic Absorption Bands From 2100 to 2240 cm^−1^

Within this region absorption bands due to N_2_O molecules containing N^15^ or O^18^ are expected. Lines due to four transitions in such isotopically substituted molecules have been identified in this study.

The band at 2201.60 cm^−1^ due to N^15^N^14^O^16^ has been previously measured by Pliva [1] but bands due to the molecules N^14^N^15^O^16^ and N^14^N^14^O^18^ found at 2164.13, 2177.66, and 2219.67 cm^−1^ have not been previously reported. The assignments for these latter three bands have been confirmed by determination of the values of *B*″ from the Δ_2_*F*″ plots. In the case of the II—II transition at 2164.13 cm^−1^ the sharp *Q* branch seems to be observable at 2164.128 cm^−1^, thus providing greater confidence in the position of the band center. Since many of the lines for these isotopic molecules were weaker than or of comparable intensity with the “hot bands” of the most abundant molecule, the identification of the lines was greatly aided by spectra obtained at 220 °K. At this temperature the intensity of the “hot band” lines is very greatly diminished. This leaves the isotopic lines as the most outstanding of the weak lines at low temperatures.

### 4.5. Discussion of Results

By means of the Ritz principle the position of some of the low-lying vibrational levels may be obtained. The bending vibration, *v*_2_, may be obtained in four different ways. Using the precise measurements of reference 4 and the recent measurements of reference 5, we find

$$v_{2}^{1}=(12^{0}0-000)-(12^{0}0-01^{1}0)=2461.998-1873.206=588.792\;{\text{cm}}^{-1}$$

$$v_{2}^{1}=(200-000)-(200-01^{1}0)=2563.341-1974.571=588.770\;{\text{cm}}^{-1}$$

$$v_{2}^{1}=(11^{1}0-000)-(11^{1}0-01^{1}0)=1880.271-1291.496=588.775\;{\text{cm}}^{-1}$$

Taking the average of the values given for the 01^1^1–000 transition in references 1 and 3, one obtains

$$v_{2}^{1}=(01^{1}1-000)-(01^{1}1-01^{1}0)=2798.308-2209.527=588.781\;{\text{cm}}^{-1}.$$

The average of these four indirect determinations, 588.780 cm^−1^, compares very favorably with the indirect measurement of Pliva [1] (588.767 cm^−1^), the four indirect measurements of Tidwell et al. [3] (588.773 cm^−1^), and the direct measurement of Lakshmi, Rao, and Nielsen [12] (588.78 cm^−1^).

The Ritz principle may also be applied to determine the values of $2v_{2}^{0}$, $2v_{2}^{2}$ and *v*_1_ as follows:

$$2v_{2}^{2}=(02^{2}1-000)-(02^{2}1-02^{2}0)=3373.184-2195.406=1177.778\;{\text{cm}}^{-1}$$

$$2v_{2}^{0}=(02^{0}1-000)-(02^{0}1-02^{0}0)=3363.997-2195.85=1168.15\;{\text{cm}}^{-1}$$

$$v_{1}=(101-000)-(101-100)=3480.854-2195.93=1284.92\;{\text{cm}}^{-1}$$

where the values for the vibration levels given by Pliva [1] and Tidwell et al. [3] are used. By using similar combinations Tidwell et al., have previously determined $2v_{2}^{2}=1177.78\;{\text{cm}}^{-1}$. McCubbin et al. [5] have recently measured $2v_{2}^{0}$ and *v*_1_ at 1168.13_4_ and 1284.90_7_ cm^−1^, respectively. These were direct measurements and should be more accurate than the indirectly obtained values given above.

The band centers for the transitions 001–000 and 01^1^1–01^1^0 have now been measured quite carefully in three different laboratories. Some idea of the absolute accuracy of these measurements may be obtained by comparing the constants derived from the measurements. We may also compare the measurements of *v*_3_ for the N^15^N^14^O^16^ molecule with those reported by Pliva [1]. Table 5 shows how closely these independent measurements agree. Although it is seen that Pliva and Fraley et al., agree on a slightly larger value for *B*_001_ than has been reported in this work, nevertheless it is felt that the values given here are probably more accurate. The present measurements extend to considerably higher values of *J* than previous measurements and as a consequence a more accurate determination of Δ*B* and Δ*D* is expected. On the other hand Fraley, Brim, and Rao probably have a more accurate value for *v*_0_ since their resolution was slightly better so that blending of low-*J* lines would have less tendency to cause errors in the derived *v*_0_. Since Pliva worked with an isotopically enriched sample, it is to be expected that his constants for N^15^N^14^O^16^ are better than those reported here.

An attempt was made to compare the results of this work with the constants given by Tidwell et al., but, as noted by Pliva [1], the agreement is not entirely satisfactory. Perhaps the measurements given here will be of value in determining the accuracy of the revised constants which Pliva is calculating.

## Figures and Tables

**Table 1:** Accurately known molecular constants used in the analysis of the *N_2_O* hands between 4.4 and 5.5 μ

*B*_000_ = 0.419010_4_ cm^−1^
*D*_000_ = 17.6×10^−8^ cm^−1^
*q* = 79.17×10^−5^ cm^−1^
*B*_010_ = 0.419572_7_ cm^−1^ (average of *c* and *d* levels)
*B*_000_(N^14^N^15^O^16^) =0.418982_1_ cm^−1^
*B*_000_(N^15^N^14^O^16^) = 0.404856_4_ cm^−1^
*B*_000_(N^14^N^14^O^18^) =0.39557_7_ cm^−1^

**Table 2:** Vibration-rotation constants describing the absorption bands of *N_2_O* in the 2000 cm^−1^ Region The limits of error given are standard deviations

Isotopic species	Assignment	*v*_0_	Δ*B*×10^+5^cm^−1^	*D′ × 10^8^	*D″ × 10^8^	Δ*D* × 10^+8^
						
N^14^N^14^O^16^	12^0^0–01^1^*^c^*0	1873.20_6_ ±0.009	−9_3_**±3	23.8	17.6	…..
	11^1^*^c^*0–000	1880.271 *±.*003	−154. _6_±0.7	17.6	17.6	−0.2±0.3
	12^2^*^c^*0–01^1^*^c^*0	}1886.03_3_ ± 00.9	− 9_6_ ± 3	$\left\{ \begin{array}{l} 13.1\\ 17.6 \end{array}\right.$	}17.6	…..
	12^2^*^d^*0–01^1^*^d^*0					
	200–01^1^*^c^*0	}1974.571 ± .003	− 397. _5_ ± 1.0	16.1	17.6	− 2.3±0.6
	200–01^1^*^d^*0					
	21^1^0–02^2^0	1988.2±.3 *— Q* branch position				
	21^1^*^d^*0–02^0^0	1997.6_5_ ± .15 *—Q* branrh position				
	02^2^*^c^*l–02^2^*^c^*0	}2195.40_6_ ± .006	− 340. _3_± 1.2	$\left\{ \begin{array}{l} 11.6\\ 17.6 \end{array}\right.$	11.6	…..
	02^2^*^d^*1–02^2^*^d^*0				17.6	…..
	02^0^l–02^0^0	2195.84_9_±.025	−336±2	23.6	23.6	0±1.4
	101–100	2195.9_3_±.04	−35_2_ ± 5	17.0	17.4	…..
	O1^1^1–01^1^0	2209.52_7_±.004	−340. _3_ ± 0.9	17.6	17.6	0.4±0.3
	001–000	2223.76_4_ ± .003	−345.6±0.3	17.4	17.6	−.26±0.05
N^14^N^15^O^16^	01^1^1–01^1^0	2164.13 ± .03	−31_5_ ± 15	17.5	17.5	…..
N^14^N^15^O^16^	001–000	2177.659	−33_0_ ± 3	17.5	17.5	…..
N^15^N^14^O^16^	001–000	2201.60_4_±.015	−33_7_ ±3	16.5	16.5	…..
N^14^N^14^O^18^	001–000	2219.67_8_ ±.02	−41_1_±10	16.5	16.5	…..

* In all cases the values of *D* given were assumed in order to obtain the best possible values of *v*_0_, Δ*B*, and Δ*D.*

** This Δ*B* value is from the average of the *c* and *d* levels of the lower state.

**Table 3:** Observed and calculated wavenumbers for the two strongest N_2_O absorption bands between 1830 and 2000 cm^−1^

*J*″	11^1^*^c^* 0–000				200–01^1^*^c^* 0				200–01^1^*^d^* 0	
	*P*		*R*		*P*		*R*		*Q*	
	Obs.	Calc.	Obs.	Calc.	Obs.	Calc.	Obs.	Calc.	Obs.	Calc.
										
0	…..	…..	1881.095	.106	…..	…..	…..	…..	…..	…..
1	…..	…..	81.929	.937	…..	…..	b1976.222	.227	…..	…..
2	b1878.595	.591	82.751	.766	…..	…..	…..	…..	…..	…..
3	b77.766	.747	83.600	.592	…..	…..	b77.832	.853	…..	…..
4	76.894	.900	…..	…..	…..	…..	b78.643	.656	…..	…..
5	76.041	.050	…..	…..	…..	…..	…..	…..	…..	…..
6	75.185	.196	…..	…..	…..	…..	b80.253	.239	…..	…..
7	…..	…..	86.865	.863	…..	…..	81.016	.020	…..	…..
8	b73.472	.480	…..	…..	…..	…..	81.790	.794	…..	…..
9	…..	…..	…..	…..	b1960.764	.769	82.559	.561	…..	…..
10	…..	…..	…..	…..	b65.880	.867	83.317	.320	…..	…..
11	…..	…..	…..	…..	64.952	.957	84.081	.073	1973.995	.995
12	…..	…..	b90.861	.882	64.034	.040	84.821	.818	…..	…..
13	…..	…..	b91.684	.677	63.127	.117	85.553	.556	73.784	.777
14	…..	…..	b92.488	.468	62.181	.186	86.287	.287	73.636	.655
15	…..	…..	93.253	.256	…..	…..	87.030	.010	73.543	.524
16	…..	…..	94.054	.041	60.302	.303	87.723	.727	73.400	.384
17	b65.590	.607	b94.850	.823	59.351	.351	…..	…..	73.218	.236
18	b64.720	.717	b95.585	.601	58.399	.392	89.127	.139	73.088	.079
19	b63.844	.824	…..	…..	57.422	.426	89.833	.834	72.920	.914
20	b62.958	.928	97.150	.149	56.448	.453	90.521	.522	72.754	.740
21	…..	…..	97.910	.918	55.465	.473	…..	…..	72.560	.557
22	61.130	.128	98.674	.684	54.494	.489	…..	…..	72.381	.366
23	60.210	.223	1899.436	.447	53.490	.493	…..	…..	72.160	.166
24	59.315	.315	b1900.190	.207	52.487	.4.92	93.222	.202	b71.986	.957
25	…..	…..	00.964	.963	51.481	.484	93.859	.855	71.746	.740
26	57.496	.491	01.715	.716	50.469	.470	94.496	.500	71.518	.514
27	56.576	.574	b02.493	.466	b49.440	.449	95.138	.138	b71.258	.280
28	55.662	.654	03.221	.213	48.423	.420	95.777	.770	b71.032	.037
29	54.739	.732	03.960	.957	47.383	.386	96.389	.394	70.787	.786
30	53.808	.806	04.689	.697	…..	…..	…..	…..	70.518	.526
31	52.884	.877	05.425	.435	…..	…..	b97.600	.621	b70.270	.258
32	51.950	.946	06.154	.169	44.234	.240	b1998.197	.224	69.987	.981
33	51.012	.012	06.883	.900	b43.171	.178			1969.700	.696
34	50.062	.074	b07.617	.627	…..	…..				
35	49.125	.134	08.348	.352	41.034	.033				
36	…..	…..	09.070	.073	39.938	.951				
37	b47.256	.245	…..	…..	38.880	.862				
38	b46.266	.296	10.517	.506	b37.783	.767				
39	…..	…..	11.220	.217	36.680	.665				
40	…..	…..	11.931	.926	35.542	.556				
41	…..	…..	12.626	.631	1934.443	.441				
42	b42.475	.472	13.345	.333						
43	41.518	.509	14.024	.032						
44	40.544	.543	b14.720	.727						
45	b39.591	.574	1915.421	.420						
46	38.604	.602								
47	…..	…..								
48	36.662	.650								
49	35.672	.670								
50	34.696	.687								
51	33.700	.701								
52	32.712	.712								
53	b1831.720	.721								

b Blended or weak lines.

**Table 4:** Wavenumbers of absorption lines for the v_1_ band of *N_2_O*

*J*	*P* Branch			*R* Branch		
	Observed wavenumber NBS	Calc.NBS	Observed wavenumber ref. 6	Observed wavenumber NBS	Calc.NBS	Observed wavenumber ref. 6
						
0	…..	…..	…..	2224.59_3_	2224.59_5_	2224.59_4_
1	b2222.90_0_	2222.92_6_	2222.94_0_	b25.41	25.41_9_	25.43_0_
2	b22.06_5_	22.08_1_	22.08_5_	b26.23_7_	26.23_6_	26.24_3_
3	b21.25_3_	21.22_9_	21.27_3_	(b)		27.05_0_
4	20.36_0_	20.37_0_	20.38_2_	27.84_5_	27.85_0_	27.84_8_
5	19.51_4_	19.50_5_	19.51_6_	28.65_0_	28.64_7_	28.63_9_
6	18.63_8_	18.63_2_	18.64_1_	b29.45_9_	29.43_6_	29.45_0_
7	17.74_5_	17.75_3_	17.75_6_	b30.22_4_	30.21_9_	30.21_8_
8	b16.83_3_	16.86_7_	16.84_0_	b30.97_5_	30.99_5_	30.97_5_
9	b15.97_5_	15.97_3_	15.98_3_	31.78_4_	31.76_3_	31.74_1_
10	b15.10_9_	15.07_3_	15.08_8_	32.54_3_	32.52_5_	32.54_8_
11	14.17_5_	14.16_6_	14.17_3_	(b)		33.30_1_
12	13.24_4_	13.25_3_	13.26_9_	b34.02_4_	34.02_8_	34.02_3_
13	12.30_4_	12.33_2_	12.33_0_	34.78_4_	34.769	34.80_6_
14	11.40_4_	11.40_5_	11.42_0_	35.52_6_	35.50_3_	35.50_9_
15	b10.46_9_	10.47_0_	10.47_8_	b36.25_3_	36.22_9_	36.24_6_
16	(b)	…..	09.52_3_	b36.94_2_	36.95_0_	36.94_5_
17	b08.58_2_	08.581	08.59_0_	b37.67_0_	37.66_3_	37.66_3_
18	09.62_5_	07.62_6_	07.62_2_	38.36_4_	38.36_9_	38.36_8_
19	06.66_9_	06.665	06.66_8_	b39.07_6_	39.06_8_	39.07_6_
20	05.68_1_	05.69_6_	05.68_7_	b39.72_8_	39.76_0_	39.76_1_
21	04.71_9_	04.72_1_	04.71_7_	40.45_4_	40.44_5_	40.44_4_
22	b03.73_7_	03.73_9_	03.73_5_	41.13_4_	41.12_3_	41.13_0_
23	b02.73_6_	02.75_0_	02.74_1_	41.78_0_	41.79_4_	41.80_5_
24	b2201.74_7_	2201.75_4_	01.75_2_	42.46_6_	42.45_8_	42.44_3_
25	…..	…..	2200.77_2_	43.12_2_	43.11_5_	43.12_0_
26	2199.72_0_	2199.74_2_	2199.73_5_	43.75_9_	43.76_5_	43.77_3_
27	98.72_6_	98.72_6_	98.73_5_	44.42_4_	44.40_9_	44.41_6_
28	97.70_7_	97.70_4_	97.69_6_	45.04_3_	45.04_5_	45.04_9_
29	96.67_7_	96.67_4_	96.66_7_	b45.66_8_	45.67_4_	45.68_8_
30	95.64_5_	95.63_8_	95.63_1_	…..	…..	46.28_9_
31	94.60_0_	94.59_4_	94.60_2_	46.89_5_	46.91_1_	46.91_8_
32	b93.52_6_	93.54_5_	93.52_8_	47.53_1_	47.51_9_	47.50_9_
33	(b)	…..	92.42_8_	48.11_4_	48.12_0_	48.11_9_
34	b91.43_2_	91.42_5_	91.43_3_	48.71_2_	48.71_4_	48.71_4_
35	b90.34_5_	90.35_5_	90.34_9_	49.30_4_	49.30_1_	49.32_8_
36	89.26_4_	89.27_8_	89.28_0_	49.88_7_	49.88_1_	49.88_9_
37	88.18_4_	88.19_4_	88.18_9_	50.46_0_	50.45_4_	50.44_9_
38	87.13_4_	87.10_4_	87.10_6_	51.03_3_	51.02_0_	51.04_7_
39	86.00_2_	86.00_7_	86.00_4_	(b)	…..	…..
40	84.91_1_	84.90_3_	84.89_0_	52.14_5_	52.13_1_	52.14_8_
41	83.79_4_	83.79_3_	83.79_0_	52.68_2_	52.67_6_	52.68_5_
42	82.66_6_	82.67_6_	82.66_7_	53.20_9_	53.21_4_	53.21_6_
43	b81.56_0_	81.55_2_	81.54_5_	(b)	…..	…..
44	(b)	…..	80.38_0_	54.23_6_	54.26_8_	54.25_3_
45	b79.30_3_	79.28_5_	79.29_3_	54.79_6_	54.78_5_	2254.79_9_
46	(b)	…..	78.14_2_	55.31_8_	55.29_5_	…..
47	76.98_2_	76.99_1_	76.98_3_	55.79_6_	55.79_7_	…..
48	75.84_0_	75.83_4_	75.83_8_	56.28_7_	56.29_3_	
49	74.67_4_	74.67_0_	74.67_2_	56.76_2_	56.78_1_	…..
50	73.49_7_	73.50_0_	73.48_8_	b57.30_0_	57.26_3_	…..
51	…..	…..	72.32_4_	b57.70_7_	57.73_7_	…..
52	71.14_4_	71.13_9_	71.13_4_	58.21_2_	58.20_5_	…..
53	b69.89_8_	…..	69.89_1_	58.66_4_	58.66_5_	…..
54	68.75_1_	68.75_2_	68.75_9_	59.13_1_	59.11_9_	…..
55	67.53_6_	67.54_9_	2167.57_9_	(b)	…..	…..
56	b66.31_8_	66.33_9_	…..	60.00_1_	60.00_4_	…..
57	(b)	…..	…..	60.41_9_	60.43_6_	…..
58	63.92_6_	63.90_0_	…..	60.88_2_	60.86_2_	…..
59	62.69_0_	62.67_0_	…..	b61.28	61.28_0_	…..
60	61.41_8_	61.43_4_	…..	61.67_5_	61.69_1_	…..
61	60.21_0_	60.19_1_	…..	62.08_2_	62.09_5_	…..
62	58.97_9_	…..	…..	62.48_9_	62.49_2_	…..
63	57.69_3_	57.68_6_	…..	(b)	…..	…..
64	56.42_2_	56.42_4_	…..	…..	…..	…..
65	55.16_8_	55.15_5_	…..	63.63_6_	63.64_0_	…..
66	53.88_3_	53.88_0_	…..	64.02_0_	64.00_9_	…..
67	…..	…..	…..	64.36_2_	64.37_1_	…..
68	51.29_5_	51.31_0_	…..	64.74_1_	64.72_5_	…..
69	b49.97_6_	50.01_5_	…..	(b)	…..	…..
70	48.74_0_	48.71_4_	…..	65.42_9_	65.41_4_	…..
71	47.36_9_	47.40_6_	…..	(b)		…..
72	46.09_7_	46.09_2_	…..	b66.08	66.07_4_	…..
73	44.73_6_	44.77_1_	…..	b66.40	66.39_3_	…..
74	b43.46	43.44_4_	…..	b66.71_3_	66.70_5_	…..
75	(b)	…..	…..	(b)	…..	…..
76	(b)	…..	…..	(b)	…..	…..
77	b39.43_8_	39.42_4_	…..	b2267.61_2_	2267.60_0_	…..
78	b2138.08_6_	2138.07_2_	…..	…..	…..	…..

b Blended or weak lines.

**Table 5:** Comparison of the results of measurements on N_2_O in different laboratories

	001–000		01^1^1–01^1^0		001–000 N^15^N^14^O^16^	
	*v*_0_	Δ*B*×10^5^	*v*_0_	Δ*B*×10^5^	*V*_0_	Δ*B*×10^5^
						
NBS (this work)	2223.764	−345.6	2209.527	−340.3	2201.604	−337
Pliva (ref. 1)	2223.754	−344.7	2209.521	−341.2	2201.605	−336.0
Fraley, Brim, and Rao (ref. 6)	2223.759	−344.6	2209.535	…..	…..	…..

## Footnotes

1 Figures in brackets indicate the literature references at the end of this paper.
